# Health of Cincinnati

**Published:** 1836

**Authors:** 


### Art. V.—Health of Cincinnati.

Our city has been entirely free from any epidemic since the cholera left it. Small-pox and varioloid have been somewhat prevalent, but they have been unusually mild except among the lowest class of emigrants. We have not seen but a single case of small-pox that proved fatal, and that was under the *mis*-management of one of the few steamers that are left among us. The subject was an industrious and temperate printer, who was doing well in the hands of an excellent physician, until a friend of the empiric prevailed upon the family to dismiss him, and employ one that would cure him at once. The result was, that under the influence of steam, No. 6, and Lobelia, the disease was speedily arrested by the death of the patient. When we saw him it was at the request of a friend, and only for the purpose of giving our opinion in relation to his case. We urged the necessity of again calling the discarded physician, but the entreaties of an ignorant female, who seemed to be completely infatuated with the quack, prevailed, and the man died. W. W.

